# Physical activity, screen exposure and sleep among students during the pandemic of COVID-19

**DOI:** 10.1038/s41598-021-88071-4

**Published:** 2021-04-20

**Authors:** Yang-feng Guo, Min-qi Liao, Wei-li Cai, Xiao-xuan Yu, Shu-na Li, Xing-yao Ke, Si-xian Tan, Ze-yan Luo, Yun-feng Cui, Qian Wang, Xu-ping Gao, Jun Liu, Yan-hua Liu, Sui Zhu, Fang-fang Zeng

**Affiliations:** 1 Department of Common Chronic Disease Control and Prevention, Health Promotion Centre for Primary and Secondary Schools of Guangzhou Municipality, Guangzhou, 510630 China; 2 Department of Epidemiology, School of Medicine, Jinan University, No.601 Huangpu Road West, Guangzhou, 510630 China; 3 Department of Child & Adolescent Psychiatry, National Clinical Research Centre for Mental Disorders & Key Laboratory of Mental Health, Ministry of Health (Peking University), Peking University Sixth Hospital (Institute of Mental Health), No.51 Huayuan Bei Road, Beijing, 100191 China; 4 Preventive Medicine Experimental Teaching Centre, Zunyi Medical University, No.6 Xuefu West Road, Zunyi, 564699 Guizhou Province China; 5 Department of Nutrition, The First Affiliated Hospital of Zhengzhou University, Zhengzhou, 450052 China; 6 Department of Medical Statistics, School of Medicine, Jinan University, No.601 Huangpu Road West, Guangzhou, 510630 China

**Keywords:** Diseases, Risk factors

## Abstract

This study aimed to determine the levels of health-related behaviours (physical activity, screen exposure and sleep status) among Chinese students from primary, secondary and high schools during the pandemic of COVID-19, as well as their changes compared with their status before the pandemic. A cross-sectional online survey of 10,933 students was conducted among 10 schools in Guangzhou, China, between 8th and 15th March, 2020. After getting the informed consent from student’s caregivers, an online questionnaire was designed and used to obtain time spending on health-related behaviours during the pandemic of COVID-19, as well as the changes compared with 3 months before the pandemic, which was completed by students themselves or their caregivers. Students were stratified by regions (urban, suburban, exurban), gender (boys and girls), and grades (lower grades of primary school, higher grades of primary schools, secondary schools and high schools). Data were expressed as number and percentages and Chi-square test was used to analyse difference between groups. Overall, the response rate of questionnaire was 95.3% (10,416/10,933). The median age of included students was 13.0 (10.0, 16.0) years and 50.1% (n = 5,219) were boys. 41.4%, 53.6% and 53.7% of total students reported less than 15 min per day in light, moderate and vigorous activities and 58.7% (n = 6,113) reported decreased participation in physical activity compared with the time before pandemic. Over 5 h of screen time spending on online study was reported by 44.6% (n = 4,649) of respondents, particular among high school students (81.0%). 76.9% of students reported increased screen time compared with the time before pandemic. Inadequate sleep was identified among 38.5% of students and the proportion was highest in high school students (56.9%). Our study indicated that, during the COVID-19 pandemic, the school closure exerted tremendous negative effects on school-aged children’s health habits, including less physical activity, longer screen exposure and irregular sleeping pattern.

## Introduction

The 2019 novel coronavirus disease (COVID-19) is an emerging disease caused by sever acute respiratory syndrome coronavirus 2 (SARS-CoV-2), firstly occurred in Wuhan, Hubei province, China, in December, 2019^1^. As of March 06, 2021, the outbreak of COVID-19 has spread to 223 countries, areas or territories, affected almost 115.3 million individuals, and caused over 2.56 million deaths worldwide^2^. School closures and other school social distance interventions were deployed rapidly across China and other 106 countries to prevent rapid transmission of the disease by March 18, 2020^3,4^. Therefore, due to restriction of group activities, team sports or playgrounds, there were more than 220 million children and adolescents, including 180 million primary and secondary students and 47 million preschool-aged children, had to be confined at home during the pandemic of COVID-19 in China^5^.

Although decreased transmission of SARS-CoV-2 has been found to be associated with the conduction of non-pharmaceutical interventions (NPIs) including school closure^6^. However, closing schools may also have downsides. Evidence suggested that student’s connection with classmates and opportunities for physical activity might greatly reduce by the enforced isolation and school closure^7,8^. Furthermore, children’s sedentary activities and screen time might expand owing to the social distancing^7^. Moreover, because the online courses were delivered through TV broadcasts or internet, school-aged children had to learn online using digital devices, which might exacerbate the overuse of media applications among children^4^. Besides, caregivers should pay attention to children’s sleeping status since evidence showed that sleep difficulties and nightmares might be attributable to the fears, uncertainties, physical and social isolation during the period staying at home^8^.

Till now, despite several studies have reported the challenges of COVID-19 pandemic for students, most of them highlighted the potential impact of the terrible COVID-19 outbreak on the mental health^9,10^, as well as its impact on education^11,12^ of university students, rather than primary, secondary and high school students. A recent study has observed worsened health-related behaviours (HRBs) among Spanish children and adolescents (aged 3 to 16 years) during the home confinement of COVID-19, including a significant reduction in physical activities, increased hours of screen exposure, as well as decreased daily consumptions of fruits and vegetables^13^. However, the self-reported data of 860 recruited children and adolescents in this study was totally obtained from 516 parents by online survey^13^, which might have significant recall bias because the physical activity, screen, and dietary data reported by children or adolescents might be different with the data obtained from their parents^14^. Further studies with larger sample size are needed to clarify the conditions of health-related behaviours among isolated students during the pandemic of COVID-19.

Hence, this study is designed to describe the status and changes in the levels of health-related behaviours (physical activity, screen exposure and sleep status) among Chinese students during school closures during the pandemic of COVID-19, in order to call attention to the potential adverse effects of school closure on student’s health.

## Methods

### Study design and participants

This descriptive cross-sectional survey was conducted in three regions (urban, suburban, and exurban) of Guangzhou, China, which represented regions with high-income, middle-income, and relatively lower-income, from 8 March 2020 to 15 March 2020. Convenience sampling was applied to obtain representative samples of primary, secondary, and high schools. The recruitment procedures included: (i) identifying potentially eligible schools via checking the records in the system including all of the schools in Guangzhou; (ii) sending an invitation letter to the targeted schools with description of the survey; (iii) telephoning the principals or deans of targeted schools to get their consent and supports; (iv) sending the link of our questionnaire to the head teachers; and (v) then sending the link to student’s caregivers by the head teachers and inviting them to participate in the survey. The exclusion criteria included: (i) students whose caregivers refused to fulfil the questionnaire; (ii) questionnaires were fulfilled within 200 seconds; and (iii) students reported BMI > 35 kg/m^2^ or <10 kg/m^2^. Finally, 10 schools, including 4 urban schools (2 primary and 2 secondary and high schools in Liwan district), 3 suburban schools (2 primary and 1 secondary and high schools in Panyu district) and 3 exurban schools (2 primary and 1 secondary and high schools in Zengcheng district), were included in the present study.

Ethics approval in accordance with the Declaration of Helsinki was obtained from the ethics committee of Health Promotion Centre for Primary and Secondary Schools of Guangzhou Municipality (No. 202001). The electronic informed consent was obtained from students or their caregivers before the survey.

### Data collection

Due to the social distancing measures and restricted movement, all the data were collected online by using a professional questionnaire App (Wenjuanxing). A structured questionnaire was designed and distributed through WeChat, the most popular instant messaging platform in China^15^. After getting the informed consent, the questionnaires for students from primary schools were mainly completed by their major caregivers (i.e., parents/other guardians), and questionnaires for students from secondary and high schools were directly fulfilled by students themselves under the supervision of their caregivers. The questionnaire was filled out anonymously. The structured questionnaire included four sections: (i) socio-demographic characteristics of the students (i.e., age, sex, school districts, grades, and monthly family income); (ii) the current status of student’s physical activity, screen exposure, and sleeping duration during the pandemic of COVID-19; and (iii) their changes compared to 3 months before the outbreak of COVID-19.

#### Physical activity

The daily duration of physical activity was assessed with the question: “During the COVID-19 pandemic, how long does you (or your child) spend in doing light (i.e., walking and jogging), moderate (i.e., brisk walking, slow jogging and cycling) and vigorous activities (i.e., running, skipping and swimming) daily on average?” The answers were categorized into four groups: “0–15 min/day”, “16–30 min/day”, “31–60 min/day”, and “> 60 min/day”. The change in physical activity was assessed with the question “Compare with the three months before the outbreak of COVID-19, is there any difference in time you (or your child) spend on daily physical activity?” The categorical answers were recoded as “Increase”, “No difference” and “Decrease”.

#### Screen exposure

The daily duration of screen exposure was assessed with the question: “How long does you (or your child) spend in using digital devices for … on average”, followed by 3 items of purposes, including “Study (attending online courses and finishing digital homework)”, “Amusement (playing computer/mobile games)” and “Leisure (chatting, reading, watching video)”. The question, “Compare with the 3 months before the outbreak of COVID-19, is there any difference in daily time you (or your child) spend in using screen-based medias (i.e., cell phone, computer or pad)?” was responded by answers involved “Appreciably increase”, “Slightly increase”, “No difference” and decrease (“Slightly increase” and “Appreciably decrease”).

#### Sleep pattern

The question, “During the COVID-19 pandemic, how long does you (or your child) sleep (including napping) daily?” was followed by the answers ranged from 5.0 to 14.0 h/day, at an interval of 0.5 h. According to the recommended of the American Academy of Sleep Medicine (AASM), sleep for 9–12 h/day and 8–10 h/day on a regular basis could promote the optimal health for children aged 6–12 years and teenagers aged 13–18 years, respectively^16^. Hence, the options included “Inadequate sleep”, “Adequate sleep” and “Excessive sleep” according to age and self-reported sleeping duration. Likewise, the questionnaire “Compare with the three months before the outbreak of COVID-19, is there any change in the amount of you (or your child) daily sleeping?” was followed by the answers “Increase”, “No difference” and “Decrease”.

### Statistical analysis

All data was entered, cleaned, and checked for missing values and outliers by two research assistants. Categorical variables were presented as number and percentages. Chi-square test was used to analyse difference between groups. All 12 grades were categorized into four groups: lower grades of primary school (grades 1–3), higher grades of primary school (grades 4–6), secondary school (grades 7–9) and high school (seniors 1–3). Stratified analyses were conducted to examine changes between groups according to school districts, sex, and grades. All data were analysed by using R-3.5.1 (R Development Core Team, Vienna, Austria) software and the statistically significant difference was identified as a two-side *P* value < 0.05.

### Ethics approval and consent to participate

Ethics approval was obtained from the ethics committee of Health Promotion Centre for Primary and Secondary Schools of Guangzhou Municipality (No. 202001). Electronic informed consent from all the participants was obtained before the survey.

## Results

### Basic characteristics of included participants

**Figure 1:**
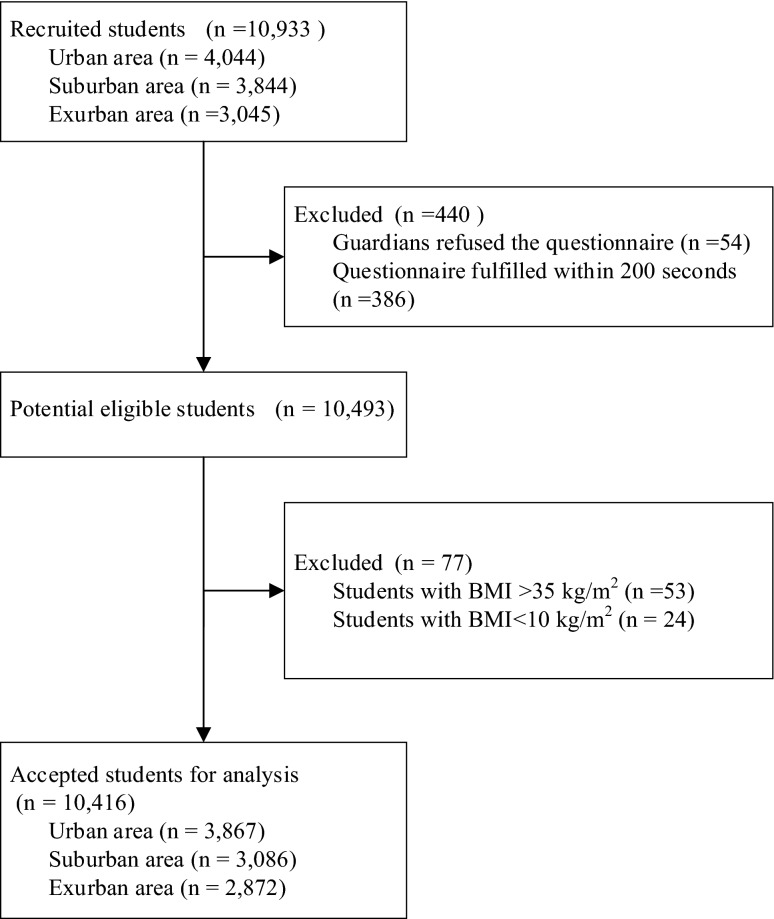
The selection procedure of participants.

The flow chart of participants selection is shown in Fig. 1. A total of 10,933 students/caregivers were successfully invited to complete the online survey. After excluding the unqualified questionnaire, 10,416 (95.3%) were included in the final analysis. The proportion of boys (5,219/10,416, 50.1%) was slightly higher than girls (5,197/10,416, 49.9%). 37.1%, 35.3% and 27.6% were from urban, suburban, and exurban areas in Guangzhou. 23.2%, 22.4%, 28.9% and 29.7% of students came from lower, higher grades of primary schools, secondary schools, and high schools. Significant differences were observed in gender, grades, and family income across regions (all *P* values < 0.05) (Table 1).

**Table 1:** Characteristics of students in various regions of Guangzhou during the COVID-19 pandemic.

	Number of participants (%)				*P* value^a^
	Overall	Urban area	Suburban area	Exurban area	
Total sample size	10,416 (100.0)	3867 (37.1)	3677 (35.3)	2872 (27.6)	
**Sex**					0.001
Boys	5219 (50.1)	1870 (48.4)	1831(49.8)	1518 (52.9)	
Girls	5197 (49.9)	1997 (51.6)	1846 50.2)	1354 (47.1)	
**Grades levels**					< 0.001
Lower grades of primary school^b^	2279 (23.2)	952 (24.6)	700 (22.7)	627 (21.8)	
Higher grades of primary school^c^	2204 (22.4)	924 (24.9)	731 (23.7)	549 (19.1)	
Secondary school	2843 (28.9)	957 (24.7)	822 (26.6)	1064 (37.0)	
High school	3090 (29.7)	1034 (26.7)	1424 38.7)	632 (22.0)	
**Family income (yuan/month)**					< 0.001
≤ 2000	523 (5.0)	145 (3.7)	111 (3.0)	267 (9.3)	
2001–5000	2462 (23.6)	753 (19.5)	738 (20.1)	971 (33.8)	
5001–10,000	2422 (23.3)	877 (22.7)	948 (25.8)	597 (20.8)	
> 10,000	1673 (16.1)	664 (17.2)	751 (20.4)	258 (9.0)	

All data are presented as frequency and its percentage.

^a^*P* values were calculated by Chi-square test.

^b^Lower grades of primary school: grades 1st to 3rd in primary school.

^c^Higher grades of primary school: grades 4th to 6th in primary school.

#### Physical activity

As shown in Table 2 and Supplementary Fig. S1, 41.4% of total students reported “16–30 min/day” for light activity and 53.6% and 53.7% of students reported only “0–15 min/day” for moderate and vigorous activities. Of three different regions, the percentages of students who participated in light, moderate and vigorous activities for less than 15 min per day were both highest in suburban area (34.6%, 56.9% and 56.1%), but for more than 60 min per day were consistently lowest in this area (5.5%, 3.5% and 4.1%).

**Table 2:** Durations of three types of student’s physical activities during the COVID-19 pandemic.

Minutes/day	Number of participants (%)											
	Overall			Urban area			Suburban area			Exurban area		
	Light activity	Moderate activity	Vigorous activity	Light activity	Moderate activity	Vigorous activity	Light activity	Moderate activity	Vigorous activity	Light activity	Moderate activity	Vigorous activity
**Overall**												
0–15	3400 (32.6)	5584 (53.6)	5596 (53.7)	1228 (31.8)	1999 (51.7)	2049 (53.0)	1272 (34.6)	2093 (56.9)	2064 (56.1)	900 (31.3)	1492 (51.9)	1483 (51.6)
16–30	4313 (41.4)	3281 (31.5)	3268 (31.4)	1587 (41.0)	1299 (33.6)	1256 (32.5)	1526 (41.5)	1084 (29.5)	1096 (29.8)	1200 (41.8)	898 (31.3)	916 (31.9)
31–60	2072 (19.9)	1155 (11.1)	1042 (10.0)	801 (20.7)	441 (11.4)	399 (10.3)	677 (18.4)	371 (10.1)	367 (10.0)	594 (20.7)	343 (11.9)	276 (9.6)
> 60	631 (6.1)	396 (3.8)	510 (4.9)	251 (6.5)	128 (3.3)	163 (4.2)	202 (5.5)	129 (3.5)	150 (4.1)	178 (6.2)	139 (4.8)	197 (6.9)
**Sex**												
Boys												
0–15	1679 (32.2)	2720 (52.1)	2638 (50.5)	562 (30.1)	963 (51.5)	940 (50.3)	650 (35.5)	1018 (55.6)	977 (53.4)	467 (30.8)	739 (48.7)	721 (47.5)
16–30	2125 (40.7)	1627 (31.2)	1645 (31.5)	780 (41.7)	614 (32.8)	622 (33.3)	747 (40.8)	542 (29.6)	546 (29.8)	598 (39.4)	471 (31.0)	477 (31.4)
31–60	1040 (19.9)	610 (11.7)	592 (11.3)	374 (20.0)	217 (11.6)	208 (11.1)	328 (17.9)	192 (10.5)	210 (11.5)	338 (22.3)	201 (13.2)	174 (11.5)
> 60	375 (7.2)	262 (5.0)	344 (6.6)	154 (8.2)	76 (4.1)	100 (5.3)	106 (5.8)	79 (4.3)	98 (5.4)	115 (7.6)	107 (7.0)	146 (9.6)
Girls												
0–15	1721 (33.1)	2864 (55.1)	2958 (56.8)	666 (33.4)	1036 (51.9)	1109 (55.5)	622 (33.7)	1075 (58.2)	1087 (58.9)	433 (32.0)	753 (55.6)	762 (56.3)
16–30	2188 (42.1)	1654 (31.8)	1623 (31.2)	807 (40.4)	685 (34.3)	634 (31.7)	779 (42.2)	542 (29.4)	550 (29.8)	602 (44.5)	427 (31.5)	439 (32.4)
31–60	1032 (19.9)	545 (10.5)	450 (8.7)	427 (21.4)	224 (11.2)	191 (9.6)	349 (18.9)	179 (9.7)	157 (8.5)	256 (18.9)	142 (10.5)	102 (7.5)
> 60	256 (4.9)	134 (2.6)	166 (3.2)	97 (4.9)	52 (2.6)	63 (3.2)	96 (5.2)	50 (2.7)	52 (2.8)	63 (4.7)	32 (2.4)	51 (3.8)
**Grades**												
Lower grades of primary school*												
0–15	675 (29.6)	1034 (45.4)	1018 (44.7)	267 (28.0)	402 (42.2)	409 (43.0)	223 (31.9)	340 (48.6)	318 (45.4)	185 (29.5)	292 (46.6)	291 (46.4)
16–30	1023 (44.9)	856 (37.6)	954 (41.9)	422 (44.3)	379 (39.8)	415 (43.6)	314 (44.9)	254 (36.3)	296 (42.3)	287 (45.8)	223 (35.6)	243 (38.8)
31–60	447 (19.6)	306 (13.4)	215 (9.4)	210 (22.1)	137 (14.4)	97 (10.2)	120 (17.1)	82 (11.7)	60 (8.6)	117 (18.7)	87 (13.9)	58 (9.3)
> 60	134 (5.9)	83 (3.6)	92 (4.0)	53 (5.6)	34 (3.6)	31 (3.3)	43 (6.1)	24 (3.4)	26 (3.7)	38 (6.1)	25 (4.0)	35 (5.6)
Higher grades of primary school**												
0–15	696 (31.6)	1092 (49.5)	1099 (49.9)	265 (28.7)	416 (45.0)	433 (46.9)	271 (37.1)	398 (54.4)	370 (50.6)	160 (29.1)	278 (50.6)	296 (53.9)
16–30	983 (44.6)	755 (34.3)	784 (35.6)	403 (43.6)	355 (38.4)	349 (37.8)	320 (43.8)	222 (30.4)	258 (35.3)	260 (47.4)	178 (32.4)	177 (32.2)
31–60	410 (18.6)	267 (12.1)	206 (9.3)	200 (21.6)	111 (12.0)	92 (10.0)	110 (15.0)	86 (11.8)	75 (10.3)	100 (18.2)	70 (12.8)	39 (7.1)
> 60	115 (5.2)	90 (4.1)	115 (5.2)	56 (6.1)	42 (4.5)	50 (5.4)	30 (4.1)	25 (3.4)	28 (3.8)	29 (5.3)	23 (4.2)	37 (6.7)
Secondary school												
0–15	785 (27.6)	1366 (48.0)	1283 (45.1)	270 (28.2)	476 (49.7)	454 (47.4)	234 (28.5)	409 (49.8)	390 (47.4)	281 (26.4)	481 (45.2)	439 (41.3)
16–30	1190 (41.9)	950 (33.4)	962 (33.8)	391 (40.9)	320 (33.4)	308 (32.2)	338 (41.1)	266 (32.4)	257 (31.3)	461 (43.3)	364 (34.2)	397 (37.3)
31–60	651 (22.9)	378 (13.3)	392 (13.8)	211 (22.0)	122 (12.7)	134 (14.0)	189 (23.0)	106 (12.9)	123 (15.0)	251 (23.6)	150 (14.1)	135 (12.7)
> 60	217 (7.6)	149 (5.2)	206 (7.2)	85 (8.9)	39 (4.1)	61 (6.4)	61 (7.4)	41 (5.0)	52 (6.3)	71 (6.7)	69 (6.5)	93 (8.7)
High school												
0–15	1244 (40.3)	2092 (67.7)	2196 (71.1)	426 (41.2)	705 (68.2)	753 (72.8)	544 (38.2)	946 (66.4)	986 (69.2)	274 (43.4)	441 (69.8)	457 (72.3)
16–30	1117 (36.1)	720 (23.3)	568 (18.4)	371 (35.9)	245 (23.7)	184 (17.8)	554 (38.9)	342 (24.0)	285 (20.0)	192 (30.4)	133 (21.0)	99 (15.7)
31–60	564 (18.3)	204 (6.6)	229 (7.4)	180 (17.4)	71 (6.9)	76 (7.4)	258 (18.1)	97 (6.8)	109 (7.7)	126 (19.9)	36 (5.7)	44 (7.0)
> 60	165 (5.3)	74 (2.4)	97 (3.1)	57 (5.5)	13 (1.3)	21 (2.0)	68 (4.8)	39 (2.7)	44 (3.1)	40 (6.3)	22 (3.5)	32 (5.1)

All data are presented as frequency and its percentage.

^a^Lower grades of primary school: grades 1st to 3rd in primary school.

^b^Higher grades of primary school: grades 4th to 6th in primary school.

When further stratification by sex and grades, we found that the percentages of those who participated in activities for less than 15 min per day were higher among girls (33.1%, 55.1%, and 56.8%) than that of boys (32.2%, 52.1%, and 50.5%). About 67.7% and 71.1% of high school students reported < 15 min/day spending in moderate and vigorous activities, which were higher than the proportions of other three grades (Table 2, Supplementary Figs. S1–S3).

**Figure 2:**
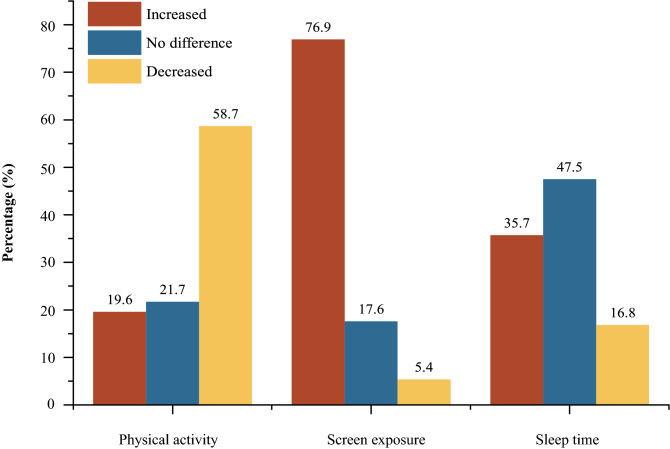
Changes in durations of physical activity, screen exposure and sleep time in students after the outbreak of COVID-19.

In Supplementary Table S1, more than half of students (58.7%) reported decreased time participating in physical activity after the outbreak of COVID-19, especially for those who came from the suburban area (64.8%; Fig. 2, Supplementary Fig. S4). After being stratified by sex and grades, we found that boys tended to report decreased time spending on physical activity compared than that of girls, particularly in suburban area (boys: 65.5% vs. girls: 63.2%). Besides, we noticed that the prevalence of students who reported decreased time on physical activity was mostly came from high school (66.9%; Supplementary Fig. S5). Especially, the proportion of high school students who reported decreased time in physical activity was highest in exurban area (69.9%).

#### Screen exposure

As shown in Table 3, 44.6% of total students reported over 5 h of screen time on online study each day during the pandemic of COVID-19, particularly among students came from urban area (48.2%). In addition, 35.0% and 49.9% of students reported 1 to 2 h of screen time for amusement and leisure, particularly in exurban area (38.4% and 51.9%) (Supplementary Fig. S6). Subgroup analyses suggested that proportion of students who reported more than 5 h for online study per day was higher among girls than that of boys (48.8% vs. 41.3%) and these proportions were highest in urban area (52.2% for girls and 43.8% for boys).

**Table 3:** Daily screen time for three purposes among students during the COVID-19 pandemic.

Hours/day	Number of participants (%)											
	Overall			Urban area			Suburban area			Exurban area		
	Study	Amusement	Leisure	Study	Amusement	Leisure	Study	Amusement	Leisure	Study	Amusement	Leisure
**Overall**												
None	84 (0.8)	3649 (35.0)	1582 (15.2)	23 (0.6)	1436 (37.1)	588 (15.2)	27 (0.7)	1293 (35.2)	550 (15.0)	34 (1.2)	920 (32.0)	444 (15.5)
0–0.5	227 (2.2)	2465 (23.7)	2880 (27.6)	54 (1.4)	919 (23.8)	1089 (28.2)	106 (2.9)	913 (24.8)	1070 (29.1)	67 (2.3)	633 (22.0)	721 (25.1)
1–2	2977 (28.6)	3646 (35.0)	5198 (49.9)	942 (24.4)	1298 (33.6)	1904 (49.2)	1177 (32.0)	1246 (33.9)	1804 (49.1)	858 (29.9)	1102 (38.4)	1490 (51.9)
3–4	2479 (23.8)	461 (4.4)	499 (4.8)	986 (25.5)	148 (3.8)	189 (4.9)	810 (22.0)	160 (44.0)	179 (4.9)	683 (23.8)	153 (5.3)	131 (4.6)
≥ 5	4649 (44.6)	195 (1.9)	257 (2.5)	1862 (48.2)	66 (1.7)	97 (2.5)	1557 (42.3)	65 (1.8)	74 (2.0)	1230 (42.8)	64 (2.2)	86 (3.0)
**Sex**												
Boys												
None	48 (0.9)	1336 (25.6)	925 (17.7)	7 (0.4)	529 (28.3)	336 (18.0)	19 (1.0)	476 (26.0)	326 (17.8)	22 (1.4)	331 (21.8)	263 (17.3)
0–0.5	137 (2.6)	1229 (23.5)	1439 (27.6)	36 (1.9)	455 (24.3)	540 (28.9)	64 (3.5)	448 (24.5)	524 (28.6)	37 (2.4)	326 (21.5)	375 (24.7)
1–2	1614 (30.9)	2177 (41.7)	2477 (47.5)	500 (26.7)	743 (39.7)	853 (45.6)	638 (34.8)	744 (40.6)	859 (46.9)	476 (31.4)	690 (45.5)	765 (50.4)
3–4	1267 (24.3)	335 (6.4)	224 (4.3)	508 (27.2)	100 (5.3)	88 (4.7)	391 (21.4)	114 (6.2)	78 (4.3)	368 (24.2)	121 (8.0)	58 (3.8)
≥ 5	2153 (41.3)	142 (2.7)	154 (3.0)	819 (43.8)	43 (2.3)	53 (2.8)	719 (39.3)	49 (2.7)	44 (2.4)	615 (40.5)	50 (3.3)	57 (3.8)
Girls												
None	36 (0.7)	2313 (44.5)	657 (12.6)	16 (0.8)	907 (45.4)	252 (12.6)	8 (0.4)	817 (44.3)	224 (12.1)	12 (0.9)	589 (43.5)	181 (13.4)
0–0.5	90 (1.7)	1236 (23.8)	1441 (27.7)	18 (0.9)	464 (23.2)	549 (27.5)	42 (2.3)	465 (25.2)	546 (29.6)	30 (2.2)	307 (22.7)	346 (25.6)
1–2	1363 (26.2)	1469 (28.3)	2721 (52.4)	442 (22.1)	555 (27.8)	1051 (52.6)	539 (29.2)	502 (27.2)	945 (51.2)	382 (28.2)	412 (30.4)	725 (53.5)
3–4	1212 (23.3)	126 (2.4)	275 (5.3)	478 (23.9)	48 (2.4)	101 (5.1)	419 (22.7)	46 (2.5)	101 (5.5)	315 (23.3)	32 (2.4)	73 (5.4)
≥ 5	2496 (48.0)	53 (1.0)	103 (2.0)	1043 (52.2)	23 (1.2)	44 (2.2)	838 (45.4)	16 (0.9)	30 (1.6)	615 (45.4)	14 (1.0)	29 (2.1)
**Grades**												
Lower grades of primary school^a^												
None	26 (1.1)	1013 (44.4)	592 (26.0)	8 (0.8)	419 (44.0)	245 (25.7)	5 (0.7)	337 (48.1)	194 (27.7)	13 (2.1)	257 (41.0)	153 (24.4)
0–0.5	106 (4.7)	565 (24.8)	584 (25.6)	27 (2.8)	229 (24.1)	255 (26.8)	60 (8.6)	172 (24.6)	191 (27.3)	19 (3.0)	164 (26.2)	138 (22.0)
1–2	1360 (59.7)	619 (27.2)	1014 (44.5)	490 (51.5)	270 (28.4)	417 (43.8)	488 (69.7)	163 (23.3)	286 (40.9)	382 (60.9)	186 (29.7)	311 (49.6)
3–4	628 (27.6)	65 (2.9)	64 (2.8)	327 (34.3)	24 (2.5)	26 (2.7)	128 (18.3)	24 (3.4)	21 (3.0)	173 (27.6)	17 (2.7)	17 (2.7)
≥ 5	159 (7.0)	17 (0.7)	25 (1.1)	100 (10.5)	10 (1.1)	9 (0.9)	19 (2.7)	4 (0.6)	8 (1.1)	40 (6.4)	3 (0.5)	8 (1.3)
Higher grades of primary school^b^												
None	18 (0.8)	770 (34.9)	347 (15.7)	9 (1.0)	345 (37.3)	152 (16.5)	5 (0.7)	232 (31.7)	110 (15.0)	4 (0.7)	193 (35.2)	85 (15.5)
0–0.5	63 (2.9)	536 (24.3)	636 (28.9)	19 (2.1)	223 (24.1)	273 (29.5)	26 (3.6)	187 (25.6)	217 (29.7)	18 (3.3)	126 (23.0)	146 (26.6)
1–2	1089 (49.4)	746 (33.8)	1078 (48.9)	331 (35.8)	297 (32.1)	442 (47.8)	482 (65.9)	251 (34.3)	354 (48.4)	276 (50.3)	198 (36.1)	282 (51.4)
3–4	739 (33.5)	116 (5.3)	108 (4.9)	363 (39.3)	49 (5.3)	41 (4.4)	177 (24.2)	42 (5.7)	40 (5.5)	199 (36.2)	25 (4.6)	27 (4.9)
≥ 5	295 (13.4)	36 (1.6)	35 (1.6)	202 (21.9)	10 (1.1)	16 (1.7)	41 (5.6)	19 (2.6)	10 (1.4)	52 (9.5)	7 (1.3)	9 (1.6)
Secondary school												
None	21 (0.7)	836 (29.4)	349 (12.3)	5 (0.5)	301 (31.5)	103 (10.8)	5 (0.6)	270 (32.8)	120 (14.6)	11 (1.0)	265 (24.9)	126 (11.8)
0–0.5	41 (1.4)	641 (22.5)	775 (27.3)	5 (0.5)	209 (21.8)	244 (25.5)	9 (1.1)	209 (25.4)	257 (31.3)	27 (2.5)	223 (21.0)	274 (25.8)
1–2	374 (13.2)	1130 (39.7)	1442 (50.7)	94 (9.8)	371 (38.8)	496 (51.8)	112 (13.6)	297 (36.1)	387 (47.1)	168 (15.8)	462 (43.4)	559 (52.5)
3–4	714 (25.1)	158 (5.6)	167 (5.9)	212 (22.2)	51 (5.3)	73 (7.6)	247 (30.0)	30 (3.6)	37 (4.5)	255 (24.0)	77 (7.2)	57 (5.4)
≥ 5	1693 (59.5)	78 (2.7)	110 (3.9)	641 (67.0)	25 (2.6)	41 (4.3)	449 (54.6)	16 (1.9)	21 (2.6)	603 (56.7)	37 (3.5)	48 (4.5)
High school												
None	19 (0.6)	1030 (33.3)	294 (9.5)	1 (0.1)	371 (35.9)	88 (8.5)	12 (0.8)	454 (31.9)	126 (8.8)	6 (0.9)	205 (32.4)	80 (12.7)
0–0.5	17 (0.6)	723 (23.4)	885 (28.6)	3 (0.3)	258 (25.0)	317 (30.7)	11 (0.8)	345 (24.2)	405 (28.4)	3 (0.5)	120 (19.0)	163 (25.8)
1–2	154 (5.0)	1151 (37.2)	1664 (53.9)	27 (2.6)	360 (34.8)	549 (53.1)	95 (6.7)	535 (37.6)	777 (54.6)	32 (5.1)	256 (40.5)	338 (53.5)
3–4	398 (12.9)	122 (3.9)	160 (5.2)	84 (8.1)	24 (2.3)	49 (4.7)	258 (18.1)	64 (4.5)	81 (5.7)	56 (8.9)	34 (5.4)	30 (4.7)
≥ 5	2502 (81.0)	64 (2.1)	87 (2.8)	919 (88.9)	21 (2.0)	31 (3.0)	1048 (73.6)	26 (1.8)	35 (2.5)	535 (84.7)	17 (2.7)	21 (3.3)

All data are presented as frequency and its percentage.

^a^Lower grades of primary school: grades 1st to 3rd in primary school.

^b^Higher grades of primary school: grades 4th to 6th in primary school.

Screen time increased with grades, particularly the time for online study and leisure (Supplementary Fig. S7). Only 7.0% of low-grade primary school students spent over 5 h/day on online study, but the proportion increased to 81.0% for students from high schools. In addition, about 27.2% of low-grade primary school students spent 1 to 2 h for amusement and 44.5% for leisure daily, but the proportions increased to 37.2% and 53.9% among high school students (Table 3).

In Supplementary Table S2, nearly 80.0% of students reported increased screen exposure after the outbreak of COVID-19. 36.0% of students reported “appreciably increased” screen time and 40.9% reported “slightly increased” screen time, particularly among students from urban area (38.4% and 39.9%; Fig. 2, Supplementary Fig. S8). In stratified analyses, slightly higher proportion of girls reported increased screen time than that of boys (78.1% vs. 75.8%). Additionally, the highest proportion of students who reported “appreciably increased screen time” was among high school students (44.7%), but the proportion of students reported “slightly increased” screen time was highest in students from lower grades of primary schools (Supplementary Fig. S9).

#### Sleep duration

As shown in Table 4, 38.5% of students reported inadequate sleep, whereas only 2.1% of students were categorized into “Excessive sleep”. This proportion was highest in those from urban area (41.5%) and lowest in suburban area (33.5%) (Supplementary Fig. S10). Higher proportion of girls reported inadequate sleep duration than that of boys (40.0% vs. 37.0%) (Supplementary Fig. S11). When stratified by grades, the highest proportion of students with inadequate sleep was reported by high school students (56.9%), while the lowest prevalence was reported by lower grades of primary school students (26.1%).

**Table 4:** Sleeping evaluation among students in various regions during the COVID-19 pandemic.

	Number of participants (%)			
	Overall	Urban area	Suburban area	Exurban area
**Overall**				
Inadequate sleep	4011 (38.5)	1606 (41.5)	1231 (33.5)	1174 (40.9)
Adequate sleep	6189 (59.4)	2190 (56.6)	2367 (64.4)	1632 (56.8)
Excessive sleep	216 (2.1)	71 (1.8)	79 (2.1)	66 (2.3)
**Sex**				
Boys				
Inadequate sleep	1932 (37.0)	738 (39.5)	590 (32.2)	604 (39.8)
Adequate sleep	3166 (60.7)	1090 (58.3)	1197 (65.4)	879 (57.9)
Excessive sleep	121 (2.3)	42 (2.2)	44 (2.4)	35 (2.3)
Girls				
Inadequate sleep	2079 (40.0)	868 (43.5)	641 (34.7)	570 (42.1)
Adequate sleep	3023 (58.2)	1100 (55.1)	1170 (63.4)	753 (55.6)
Excessive sleep	95 (1.8)	29 (1.5)	35 (1.9)	31 (2.3)
**Grades**				
Lower grades of primary school^a^				
Inadequate sleep	594 (26.1)	218 (22.9)	159 (22.7)	217 (34.6)
Adequate sleep	1664 (73.0)	723 (75.9)	533 (76.1)	408 (65.1)
Excessive sleep	21 (0.9)	11 (1.2)	8 (1.1)	2 (0.3)
Higher grades of primary school^b^				
Inadequate sleep	836 (37.9)	337 (36.5)	249 (34.1)	250 (45.5)
Adequate sleep	1354 (61.4)	582 (63.0)	475 (65.0)	297 (54.1)
Excessive sleep	14 (0.6)	5 (0.5)	7 (1.0)	2 (0.4)
Secondary school				
Inadequate sleep	822 (28.9)	303 (31.7)	182 (22.1)	337 (31.7)
Adequate sleep	1893 (66.6)	609 (63.6)	606 (73.7)	678 (63.7)
Excessive sleep	128 (4.5)	45 (4.7)	34 (4.1)	49 (4.6)
High school				
Inadequate sleep	1759 (56.9)	748 (72.3)	641 (45.0)	370 (58.5)
Adequate sleep	1278 (41.4)	276 (26.7)	753 (52.9)	249 (39.4)
Excessive sleep	53 (1.7)	10 (1.0)	30 (2.1)	13 (2.1)

All data are presented as frequency and its percentage.

^a^Lower grades of primary school: grades 1st to 3rd in primary school.

^b^Higher grades of primary school: grades 4th to 6th in primary school.

As shown in Supplementary Table S3 and Fig. 2, 35.7% of students reported increased sleeping duration since the outbreak of COVID-19, whereas 16.8% of students reported decreased sleep duration. The highest proportions of students with increased and decreased sleeping duration were observed in suburban area (41.9%) and exurban area (21.2%) (Supplementary Fig. S12). Generally, the proportion of students who reported increased sleep duration decreased with increased grades (lower grades of primary school students: 40.4% vs. high school students: 31.5%), whereas inverse trend was observed for reduced sleeping time (lower grades of primary school students: 11.6% vs. high school students: 24.7%) (Supplementary Fig. S13).

## Discussion

In this cross-sectional study, we observed significant decreased time spent on physical activity, longer screen time, and abnormal sleeping duration among primary, secondary and high school students during or compared with three months before the outbreak of COVID-19. Of note, we noticed that these unhealthy lifestyles occurred more frequently among students in higher grades and those from urban area.

Recently, Moore et al.^17^ found that less than 5.0% of children and only 0.6% of youths across Canada met the combined movement behaviour guidelines during the initial period of COVID-19 crisis. They also reported more sedentary and screen-based activities and longer sleeping time among children and adolescents compared with before the school closure^17^. Another study conducted in Spain consistently reported that school closure might worsen most health-related behaviours (reduced physical activity and increased screen time and sleep time among school-aged children and adolescents^13^. However, both two studies were totally based on parent-reported data, the possibility of social desirability and recall bias could affect their findings. An international study conducted among 726 adolescents aged 16–19 years old from Europe (Italy and Spain) and Latin America (Brazil, Chile, and Colombia) reported reductions of physical activity during the pandemic^18^. Nevertheless, this study was based on a convenience sample from different countries and their findings could not generalize to the entire population from those places.

During the pandemic of COVID-19, the confinement at home with reduced opportunities for physical activity, thus the levels of physical activity significantly decreased among students^7^. Participations in team sports and activity at recess might benefit to the well-being of students and promote the prosocial behaviour and counteract disaffection in adolescents^19,20^. Physical education classes might play an important role in helping youth attain sufficient levels of physical activity during school time. However, the access to physical activity and the all these activity-related benefits would be diminished due to the prolonged school closure^21^. Most of children in urban area were forced to stay at home and their engagement in activities outdoor, such as in parks and playgrounds, would be limited by their caregivers since they could not ensure that these places were clean and safe enough^7^. Of note, the physical activity engagement among high school students might be lower than that of lower grade students due to their heavier educational burden. Previous studies consistently showed that student’s physical inactivity might increase with age and education levels^22,23^. Physical activity may appeal more to boys rather than girls^22^, exacerbating the sex disparity of self-reported decline in physical activity. Insufficient physical activity might affect the growth and development of children and adolescents, leading to several adverse consequences such as elevated insulin and blood lipids level^24^, obesity^25^, coronary heart disease and cancers^26^, or mental outcomes such as poor social behaviour^20^ and depression^27^. Thus, maintaining regular physical activity in a safe environment need to be promoted for healthy living during the crisis^28^.

In our survey, we found that 42.3–48.2% of total students reported more than 5 h per day on on-line study via digital devices, especially among high school students who had higher pressure for entrance to a better college^29^. Compared to lower grade students, they had to spend longer time on attending on-line classes, finishing homework, and taking the examination through the digital devices during the school closure. Without the stress-reduced effects of physical education participation^30^ and peer connectedness^29^, the heavy workload might associated with greater distress and negative mood^31^, then might exacerbate or trigger underlying stress vulnerability among high school students during the suspend of classes.

Over 50% of students spent over one hour per day in amusement and leisure. Under the home confinement, students might spend more time in using screen-based media to pass the time^32^. Without adult supervision, children might be exposed to an open-ended periods of screen time due to less regulation or restriction^33^, especially for the high school students who had limited opportunities to play video games or watch TV in school. The screen time for majority of students exceeded the screen-based recommendations of less than 2 h screening time per day^34^. A series of symptoms (eye fatigue, blurred vision, or eye dryness) could be caused by excessive computer screen exposure^35^, and myopia was the primary concern after long screen time^36^. Compared those exposing to screen devices < 2 h/day, using screen devices over 6 h/day induced about doubled risk of suffering myopia^36^. In addition, excessive and addictive use of digital media might be associated with obesity^37^, reduced bone density^35^, poor sleep^38^, and even damaged psychosocial health in youths^39^.

Our study also indicated that children’s sleep become irregular during the crisis. The reason might be due to that, the students were given more freedom to stay up late at night and wake up late in the morning during the less-structed days in response to the school closure. In addition, increased risk of irregular sleep pattern occurred when students were out of school^4^. During the school days, most of students had to go to bed earlier and wake up on time to meet the need of attending school^21^. However, previous study provided supporting evidence that child’s bed/wake-times might be later in weekend than that in weekends^40^. Irregular sleeping duration might worsen children’s physical and mental conditions^41^, as well as academic performance^42^. Reduced sleep might also exert negative effects on children’s health, such as overweight/obesity and depressive symptoms^43,44^.

The strength of the present epidemiologic study is the relatively large sample size. However, the limitations of this study should be acknowledged. Firstly, conducting through an online questionnaire during the pandemic of COVID-19, this survey was inevitable to subject to nonresponse bias. Secondly, in order to ensure the timeliness and conciseness of the questionnaire survey, the authors conducted this survey even though some specific questions were not specifically validated. Thirdly, in order to collect the valuable information as soon as possible in this critical period, as well as maintaining the compliance of participants, there might be some important factors have not been included in our study aside from the basic demographic information such as gender, age and grades. Fourthly, the quality of the respondents was determined by student’s grades, thus the recall bias might be caused by the inconsistent responders. We tried to reduce this bias through suggesting caregivers to supervise and explanate each question for students from all grades during the fulfilling of our questionnaire. Fifthly, there might be selection bias because all the included schools were not randomly selected; however, we included a relatively large number of students from primary, secondary, and high schools from three different districts to minimize the potential influence of sampling. Finally, the samples were only recruited from schools in Guangzhou, one of the most developed regions in China. Our findings generalized to other cities or countries should be with caution.

## Conclusion

Taken together, findings from this survey revealed that the school closure during the COVID-19 pandemic might have several adverse impacts on the healthy lifestyle habits of school-aged children and adolescents, including decreased engagement in physical activity, longer screen exposure and irregular sleeping duration. However, according to guidance recommended by the National Health Commission of the People’s Republic of China during the pandemic of COVID-19^45^, school-aged children should do 2 h or more of outdoor exercises and 30 min recess physical activity daily. Daily screen time for online learning should be limited within 2.5 h for students from primary schools and 4 h for students from secondary and high schools, and screen time for other purposes should be limited to within 1 h per day. Students from primary, secondary, and high schools should sleep no less than 10, 9 and 8 h daily, respectively. Hence, close attention and great efforts are required to address these issues timely under the control of this pandemic, including offering more opportunities for physical activity, providing guidelines to limit screen exposure and to maintain regular sleeping pattern among school-aged children and adolescents.

## Supplementary Information

Supplementary Information.

## Data Availability

The datasets during and/or analysed during the current study available from the corresponding author on reasonable request.
