# Supplementary material for: Physical activity, screen exposure and sleep among students during the pandemic of COVID-19
Source: Sci Rep. 2021 Apr 20;11:8529. doi: 10.1038/s41598-021-88071-4 (PMC8058040; doi:10.1038/s41598-021-88071-4)
Supplement: Supplementary file 1 — Supplementary Information. [file 41598_2021_88071_MOESM1_ESM.docx]

**Supplementary information**

**Physical activity, screen exposure and sleep among students during the pandemic of COVID-19**

Yang-feng Guo ^1, #^, Min-qi Liao ^2^ , Wei-li Cai ^1^, Xiao-xuan Yu ^2^, Shu-na Li ^2^, Xing-yao Ke ^2^, Si-xian Tan ^2^, Ze-yan Luo ^2^, Yun-feng Cui ^2^, Qian Wang ^2^, Xu-ping Gao ^3^, Jun Liu ^4^, Yan-hua Liu ^5^, Sui Zhu ^6^, Fang-fang Zeng ^2,*^.

**Table captions**

**Supplementary Table S1.** Changes in duration of physical activity among students in various regions of Guangzhou during the COVID-19 pandemic.

**Supplementary Table S2.** Changes in daily screen exposure among students in various regions of Guangzhou during the COVID-19 pandemic.

**Supplementary Table S3.** Changes in sleeping duration among students in various regions of Guangzhou during the COVID-19 pandemic.

**Figure legends**

**Supplementary Figure S1.** Daily durations of three types of physical activities among students of different regions during COVID-19 pandemic.

**Supplementary Figure S2.** Daily durations of three types of physical activities among students by sex categories during COVID-19 pandemic.

**Supplementary Figure S3.** Daily durations of three types of physical activities among students in different grade categories during COVID-19 pandemic.

**Supplementary Figure S4.** Changes in durations of physical activity among students of different grade categories during COVID-19 pandemic.

**Supplementary Figure S5.** Changes in durations of physical activity among students of different grade categories during COVID-19 pandemic.

**Supplementary Figure S6.** Daily screen time for three purposes among students in various regions during COVID-19 pandemic.

**Supplementary Figure S7.** Daily screen time for three purposes among students of different grade categories during COVID-19 pandemic.

**Supplementary Figure S8.** Changes in daily screen time among students in various regions during COVID-19 pandemic.

**Supplementary Figure S 9.** Changes in daily screen time among students of different grade categories during COVID-19 pandemic.

**Supplementary Figure S10.** Sleeping evaluation among students in various regions during COVID-19 pandemic.

**Supplementary Figure S11.** Sleeping evaluation among students by sex during COVID-19 pandemic.

**Supplementary Figure S12.** Changes in sleeping duration among students in various regions during COVID-19 pandemic.

**Supplementary Figure S13.** Changes in sleeping duration among students of different grade categories during COVID-19 pandemic.

**Supplementary Table S1**. Changes in duration of physical activity among students in various regions of Guangzhou during the COVID-19 pandemic.

|  | **Number of participants (%)** | | | |
| --- | --- | --- | --- | --- |
|  | **Overall** | **Urban area** | **Suburban area** | **Exurban area** |
| **Overall** |  |  |  |  |
| Increased | 2038 (19.6) | 827 (21.4) | 567 (15.4) | 644 (22.4) |
| No difference | 2265 (21.7) | 825 (21.3) | 727 (19.8) | 713 (24.8) |
| Decreased | 6113 (58.7) | 2215 (57.3) | 2383 (64.8) | 1515 (52.8) |
| **Sex** |  |  |  |  |
| ***Boys*** |  |  |  |  |
| Increased | 971 (19.6) | 393 (21.0) | 242 (15.6) | 336 (22.1) |
| No difference | 1078 (21.8) | 397 (21.2) | 294 (18.9) | 387 (25.5) |
| Decreased | 2894 (58.5) | 1080 (57.8) | 1019 (65.5) | 795 (52.4) |
| ***Girls*** |  |  |  |  |
| Increased | 994 (20.4) | 434 (21.7) | 252 (16.5) | 308 (22.7) |
| No difference | 1066 (21.8) | 428 (21.4) | 312 (20.4) | 326 (24.1) |
| Decreased | 2822 (57.8) | 1135 (56.8) | 967 (63.2) | 720 (53.2) |
| **Grades** |  |  |  |  |
| ***Lower grades of primary school ^a^*** | | |  |  |
| Increased | 551 (24.2) | 242 (25.4) | 145 (20.7) | 164 (26.2) |
| No difference | 466 (20.4) | 189 (19.9) | 121 (17.3) | 156 (24.9) |
| Decreased | 1262 (55.4) | 521 (54.7) | 434 (62.0) | 307 (49.0) |
| ***Higher grades of primary school ^b^*** | | |  |  |
| Increased | 475 (21.6) | 244 (26.4) | 111 (15.2) | 120 (21.9) |
| No difference | 448 (20.3) | 205 (22.2) | 113 (15.5) | 130 (23.7) |
| Decreased | 1281 (58.1) | 475 (51.4) | 507 (69.4) | 299 (54.5) |
| ***Secondary school*** | | |  |  |
| Increased | 638 (22.4) | 200 (20.9) | 139 (16.9) | 299 (28.1) |
| No difference | 702 (24.7) | 224 (23.4) | 180 (21.9) | 298 (28.0) |
| Decreased | 1503 (52.9) | 533 (55.7) | 503 (61.2) | 467 (43.9) |
| ***High school*** | | |  |  |
| Increased | 374 (12.1) | 141 (13.6) | 172 (12.1) | 61 (9.7) |
| No difference | 649 (21.0) | 207 (20.0) | 313 (22.0) | 129 (20.4) |
| Decreased | 2067 (66.9) | 686 (66.3) | 939 (65.9) | 442 (69.9) |

Note: All data are presented as frequency and its percentage.

^a^ Lower grades of primary school: grades 1^st^ to 3^rd^ in primary school.

^b^ Higher grades of primary school: grades 4^th^ to 6^th^ in primary school.

**Supplementary Table S2.** Changes in daily screen exposure among students in various regions of Guangzhou during the COVID-19 pandemic.

|  | **Number of participants (%)** | | | |
| --- | --- | --- | --- | --- |
|  | **Overall** | **Urban area** | **Suburban area** | **Exurban area** |
| **Overall** |  |  |  |  |
| Appreciably increased | 3752 (36.0) | 1485 (38.4) | 1385 (37.7) | 882 (30.7) |
| Slightly increased | 4264 (40.9) | 1544 (39.9) | 1539 (41.9) | 1181 (41.1) |
| No difference | 1834 (17.6) | 636 (16.4) | 596 (16.2) | 602 (21.0) |
| Decreased | 566 (5.4) | 202 (5.2) | 157 (4.3) | 207 (7.2) |
| **Sex** |  |  |  |  |
| ***Boys*** |  |  |  |  |
| Appreciably increased | 1859 (35.6) | 692 (37.0) | 710 (38.8) | 457 (30.1) |
| Slightly increased | 2098 (40.2) | 749 (40.1) | 735 (40.1) | 614 (40.4) |
| No difference | 945 (18.1) | 319 (17.1) | 299 (16.3) | 327 (21.5) |
| Decreased | 317 (6.1) | 110 (5.9) | 87 (4.8) | 120 (7.9) |
| ***Girls*** |  |  |  |  |
| Appreciably increased | 1893 (36.4) | 793 (39.7) | 675 (36.6) | 425 (31.4) |
| Slightly increased | 2166 (41.7) | 795 (39.8) | 804 (43.6) | 567 (41.9) |
| No difference | 889 (17.1) | 317 (15.9) | 297 (16.1) | 275 (20.3) |
| Decreased | 249 (4.8) | 92 (4.6) | 70 (3.8) | 87 (6.4) |
| **Grades** |  |  |  |  |
| ***Lower grades of primary school ^a^*** | |  |  |  |
| Appreciably increased | 780 (34.2) | 349 (36.7) | 228 (32.6) | 203 (32.4) |
| Slightly increased | 1091 (47.9) | 453 (47.6) | 349 (49.9) | 289 (46.1) |
| No difference | 316 (13.9) | 113 (11.9) | 99 (14.1) | 104 (16.6) |
| Decreased | 92 (4.0) | 37 (3.9) | 24 (3.4) | 31 (4.9) |
| ***Higher grades of primary school ^b^*** | |  |  |  |
| Appreciably increased | 822 (37.3) | 348 (37.7) | 303 (41.5) | 171 (31.1) |
| Slightly increased | 912 (41.4) | 369 (39.9) | 306 (41.9) | 237 (43.2) |
| No difference | 341 (15.5) | 144 (15.6) | 94 (12.9) | 103 (18.8) |
| Decreased | 129 (5.9) | 63 (6.8) | 28 (3.8) | 38 (6.9) |
| ***Secondary school*** | |  |  |  |
| Appreciably increased | 769 (27.0) | 257 (26.9) | 275 (33.5) | 237 (22.3) |
| Slightly increased | 1157 (40.7) | 408 (42.6) | 304 (37.0) | 445 (41.8) |
| No difference | 707 (24.9) | 234 (24.5) | 193 (23.5) | 280 (26.3) |
| Decreased | 210 (7.4) | 58 (6.1) | 50 (6.1) | 102 (9.6) |
| ***High school*** |  |  |  |  |
| Appreciably increased | 1381 (44.7) | 531 (51.4) | 579 (40.7) | 271 (42.9) |
| Slightly increased | 1104 (35.7) | 314 (30.4) | 580 (40.7) | 210 (33.2) |
| No difference | 470 (15.2) | 145 (14.0) | 210 (14.7) | 115 (18.2) |
| Decreased | 135 (4.4) | 44 (4.3) | 55 (3.9) | 36 (5.7) |

Note: All data are presented as frequency and its percentage.

^a^ Lower grades of primary school: grades 1^st^ to 3^rd^ in primary school.

^b^ Higher grades of primary school: grades 4^th^ to 6^th^ in primary school.

**Supplementary Table S3.** Changes in sleeping duration among students in various regions of Guangzhou during the COVID-19 pandemic.

|  | **Number of participants (%)** | | | |
| --- | --- | --- | --- | --- |
|  | **Overall** | **Urban area** | **Suburban area** | **Exurban area** |
| **Overall** |  |  |  |  |
| Increased | 3717 (35.7) | 1248 (32.3) | 1541 (41.9) | 928 (32.3) |
| No difference | 4945 (47.5) | 1955 (50.6) | 1655 (45.0) | 1335 (46.5) |
| Decreased | 1754 (16.8) | 664 (17.2) | 481 (13.1) | 609 (21.2) |
| **Sex** |  |  |  |  |
| ***Boys*** |  |  |  |  |
| Increased | 1857 (35.6) | 601 (32.1) | 760 (41.5) | 496 (32.7) |
| No difference | 2526 (48.4) | 979 (52.4) | 828 (45.2) | 719 (47.4) |
| Decreased | 836 (16.0) | 290 (15.5) | 243 (13.3) | 303 (20.0) |
| ***Girls*** |  |  |  |  |
| Increased | 1869 (36.0) | 647 (32.4) | 781 (42.3) | 432 (31.9) |
| No difference | 2419 (46.5) | 976 (48.9) | 827 (44.8) | 616 (45.5) |
| Decreased | 918 (17.7) | 374 (18.7) | 238 (12.9) | 306 (22.6) |
| **Grades** |  |  |  |  |
| ***Lower grades of primary school ^a^*** | | |  |  |
| Increased | 870 (38.2) | 356 (37.4) | 286 (40.9) | 228 (36.4) |
| No difference | 1145 (50.2) | 501 (52.6) | 328 (46.9) | 316 (50.4) |
| Decreased | 264 (11.6) | 95 (10.0) | 86 (12.3) | 83 (13.2) |
| ***Higher grades of primary school ^b^*** | | |  |  |
| Increased | 891 (40.4) | 362 (39.2) | 321 (43.9) | 208 (37.9) |
| No difference | 1073 (48.7) | 470 (50.9) | 335 (45.8) | 268 (48.8) |
| Decreased | 240 (10.9) | 92 (10.0) | 75 (10.3) | 73 (13.3) |
| ***Secondary school*** | | |  |  |
| Increased | 984 (34.6) | 296 (30.9) | 324 (39.4) | 364 (34.2) |
| No difference | 1371 (48.2) | 486 (50.8) | 392 (47.7) | 493 (46.3) |
| Decreased | 488 (17.2) | 175 (18.3) | 106 (12.9) | 207 (19.5) |
| ***High school*** | | |  |  |
| Increased | 972 (31.5) | 234 (22.6) | 610 (42.8) | 128 (20.3) |
| No difference | 1356 (43.9) | 498 (48.2) | 600 (42.1) | 258 (40.8) |
| Decreased | 762 (24.7) | 302 (29.2) | 214 (15.0) | 246 (38.9) |

Note: All data are presented as frequency and its percentage.

^a^ Lower grades of primary school: grades 1^st^ to 3^rd^ in primary school.

^b^ Higher grades of primary school: grades 4^th^ to 6^th^ in primary school.


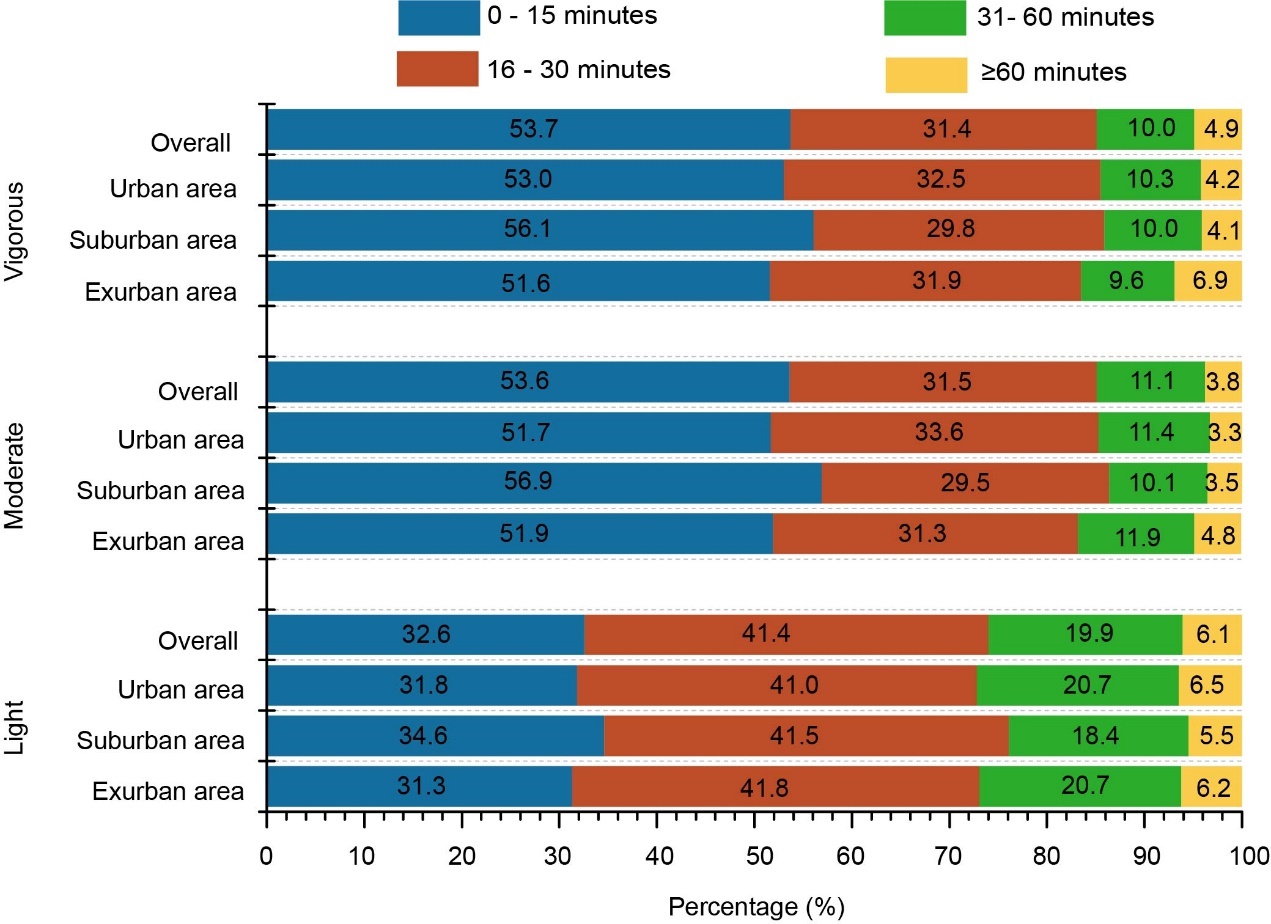


**Supplementary Figure S1.** Daily durations of three types of physical activities among students of different regions during COVID-19 pandemic.


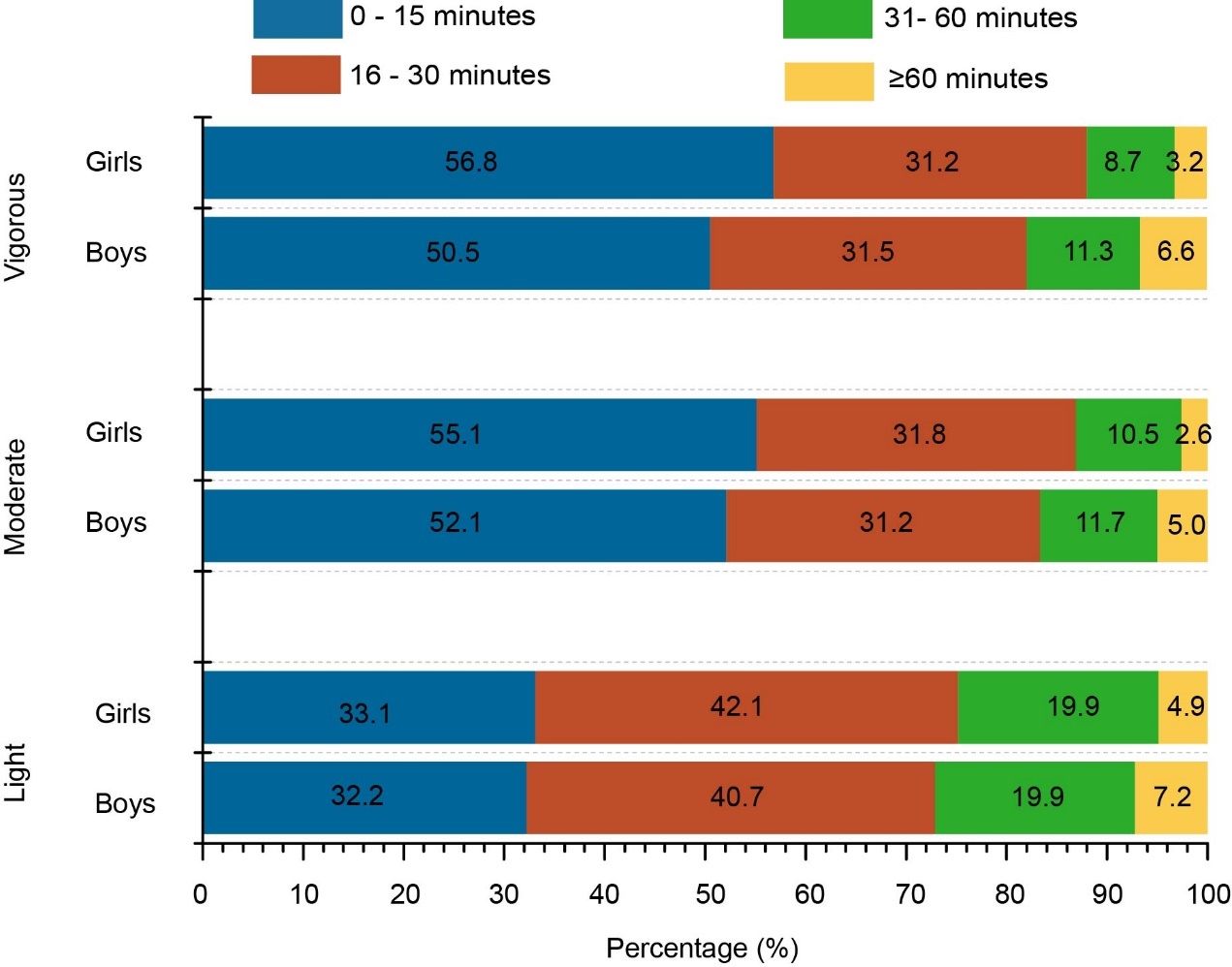


**Supplementary Figure S2.** Daily durations of three types of physical activities among students by sex categories during COVID-19 pandemic.


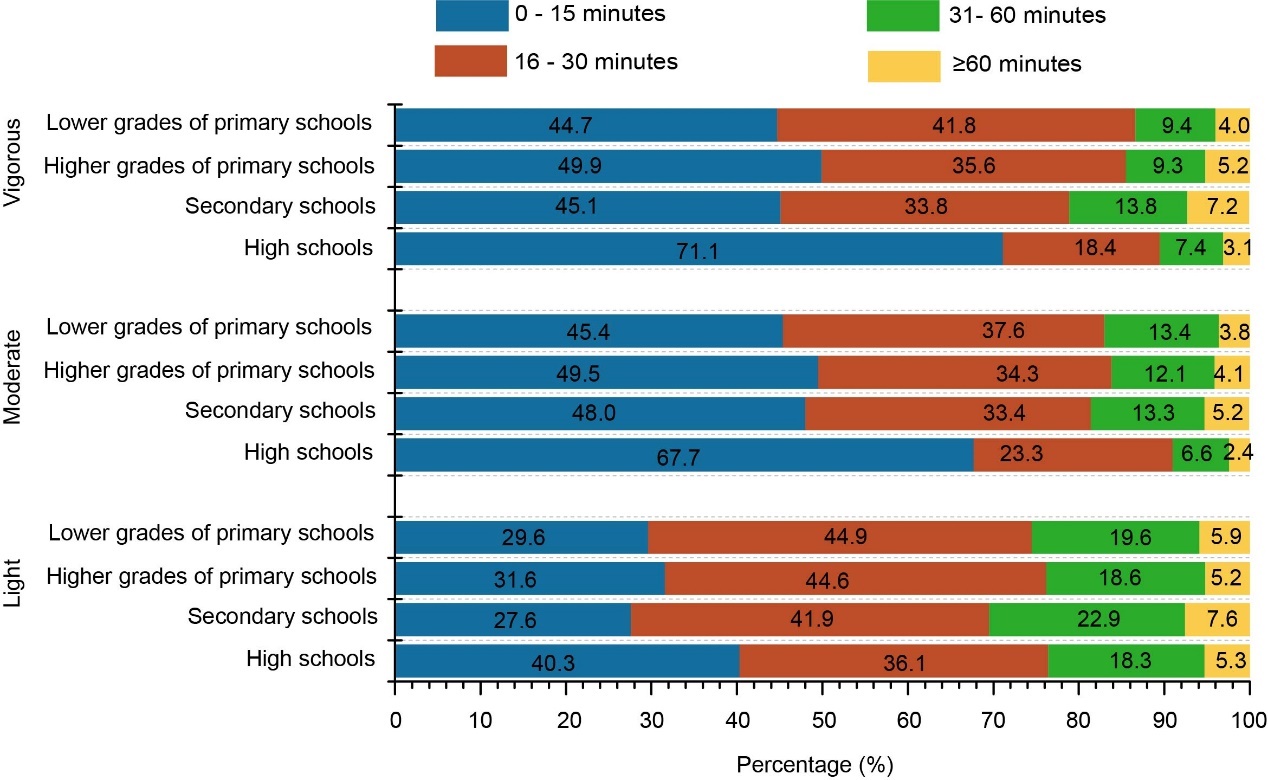


**Supplementary Figure S3.** Daily durations of three types of physical activities among students in different grade categories during COVID-19 pandemic.


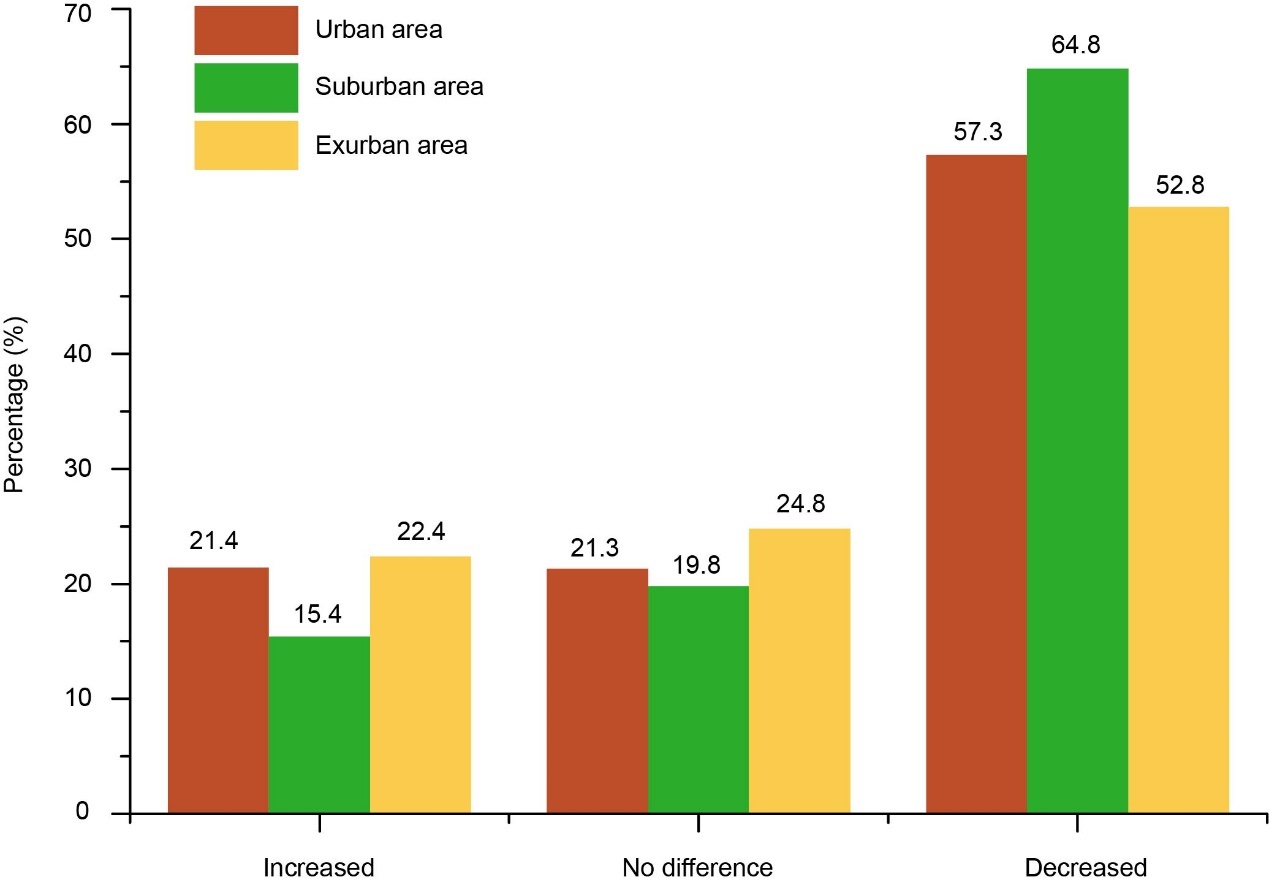


**Supplementary Figure S4.** Changes in durations of physical activity among students of different grade categories during COVID-19 pandemic.


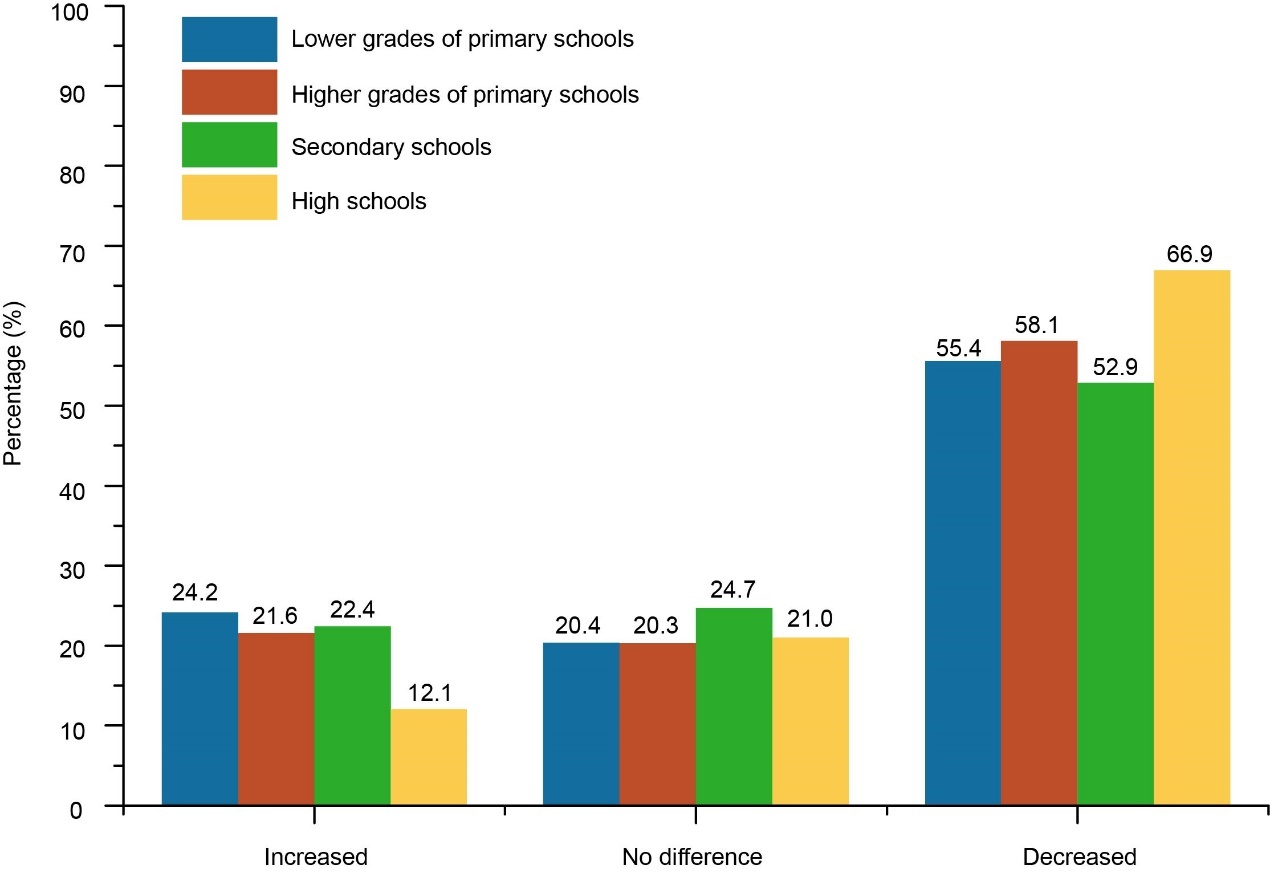


**Supplementary Figure S5.** Changes in durations of physical activity among students of different grade categories during COVID-19 pandemic.


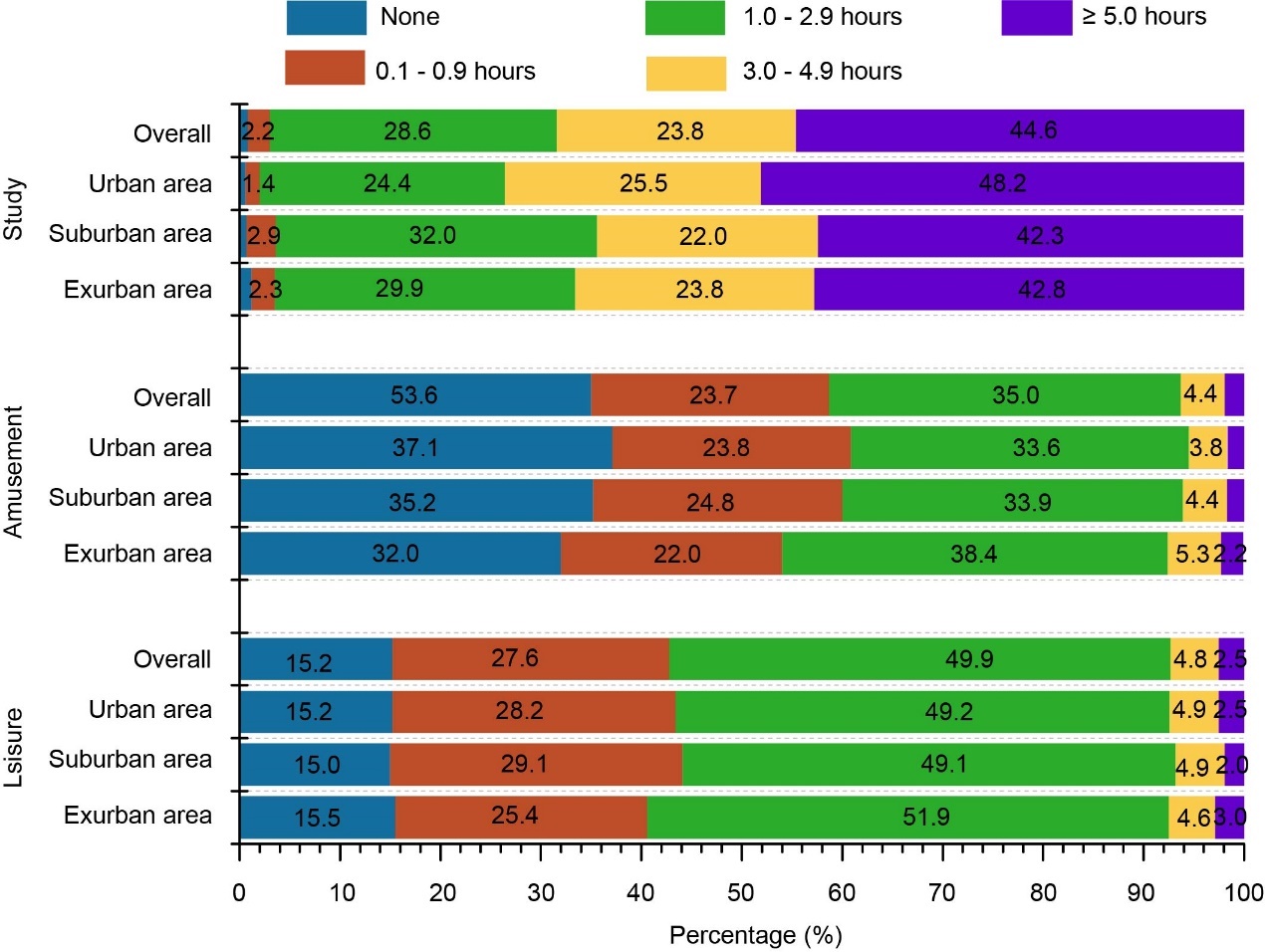


**Supplementary Figure S6.** Daily screen time for three purposes among students in various regions during COVID-19 pandemic.


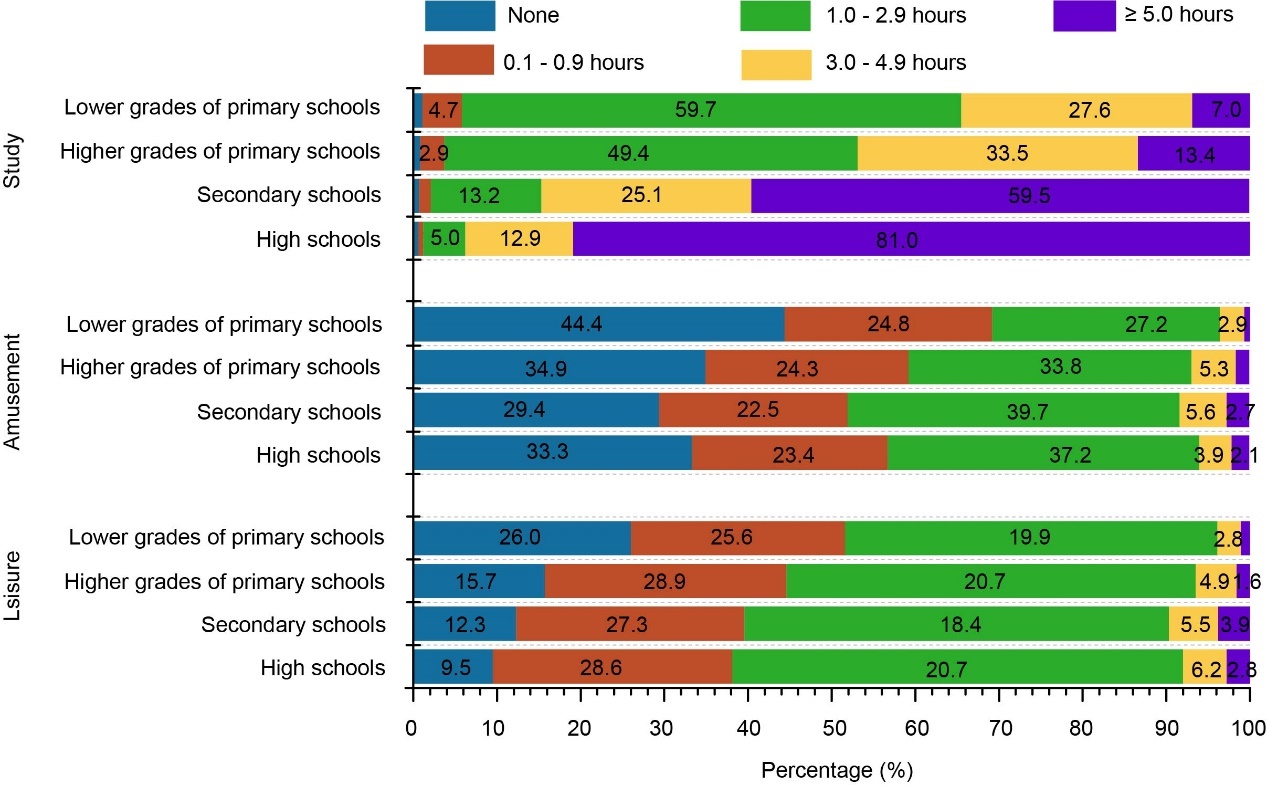


**Supplementary Figure S7.** Daily screen time for three purposes among students of different grade categories during COVID-19 pandemic.


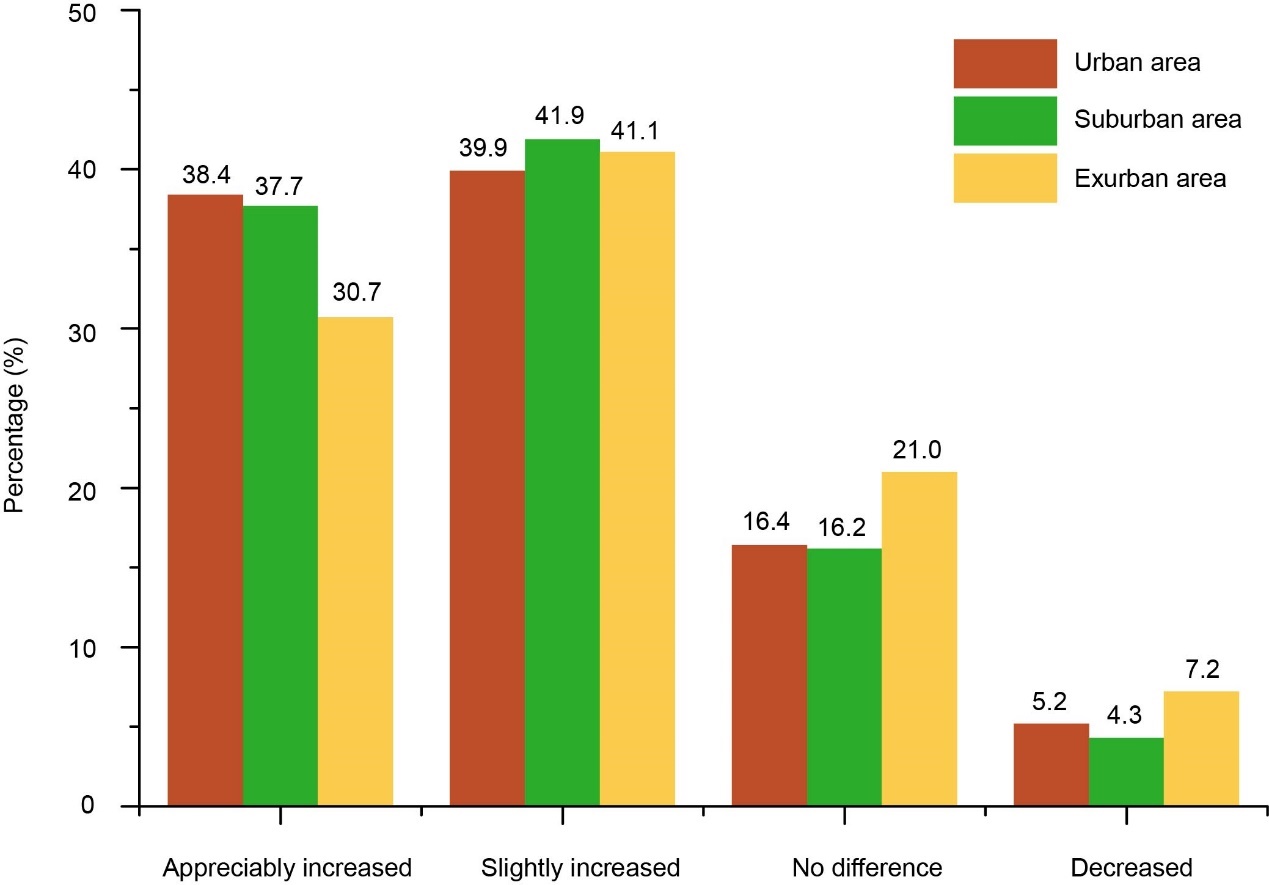


**Supplementary Figure S8.** Changes in daily screen time among students in various regions during COVID-19 pandemic.


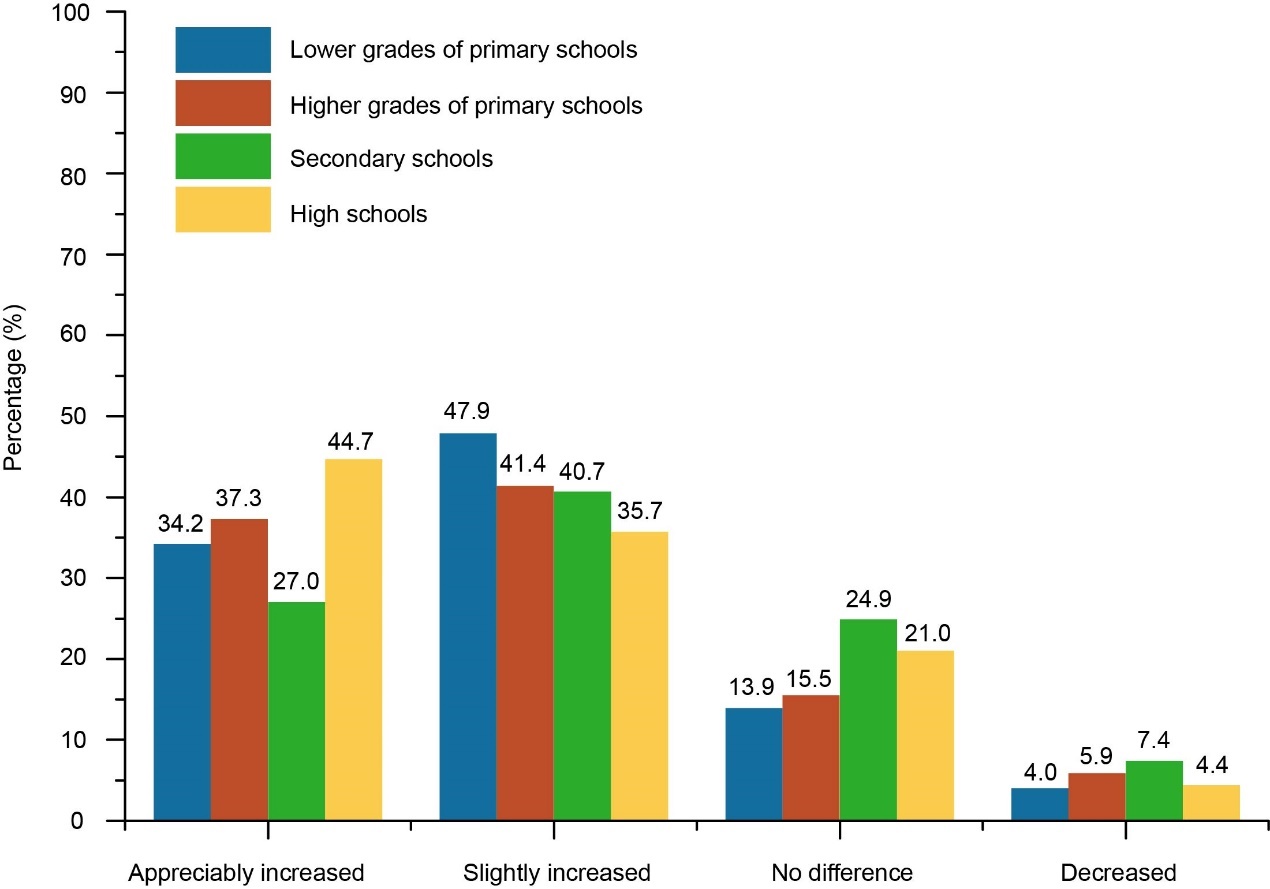


**Supplementary Figure S9.** Changes in daily screen time among students of different grade categories during COVID-19 pandemic.


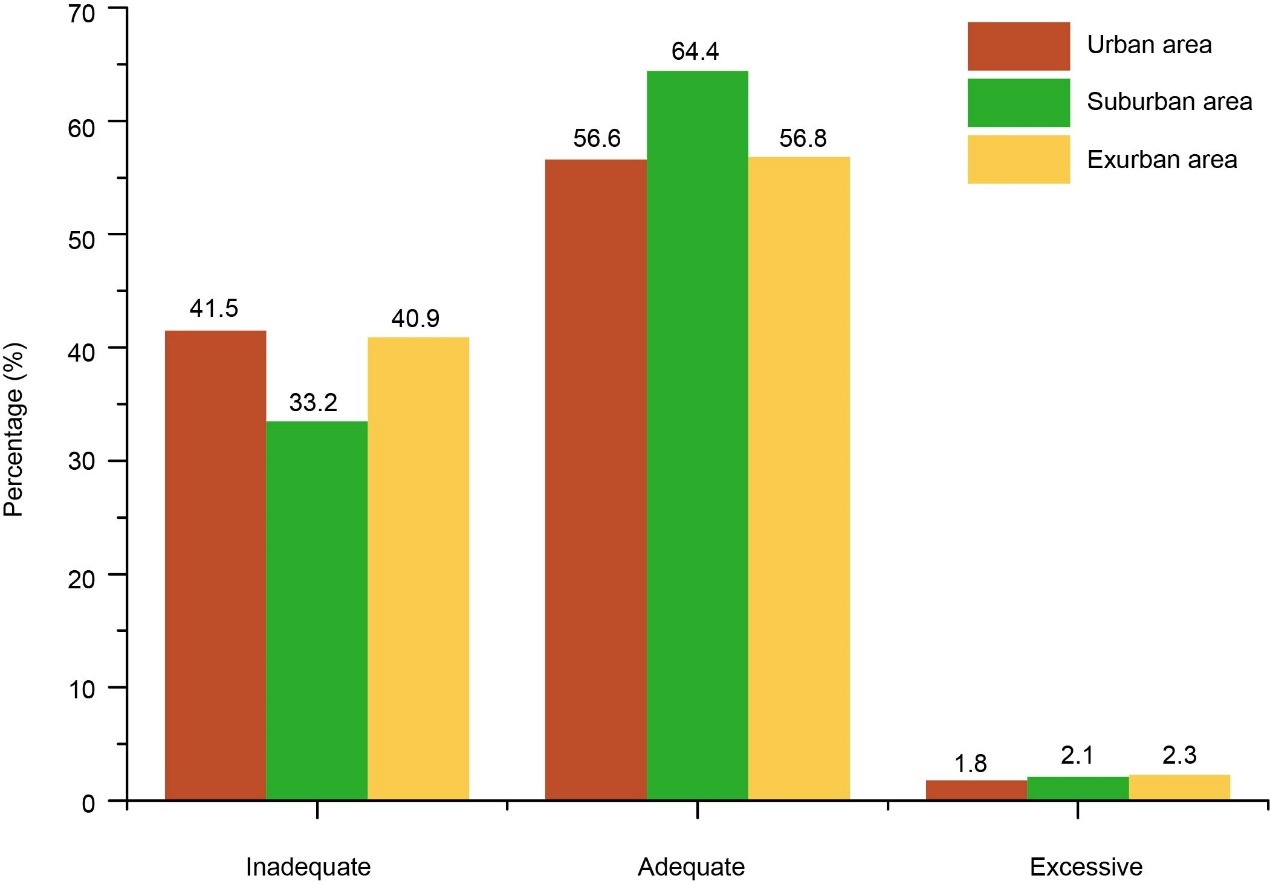


**Supplementary Figure S10.** Sleeping evaluation among students in various regions during COVID-19 pandemic.


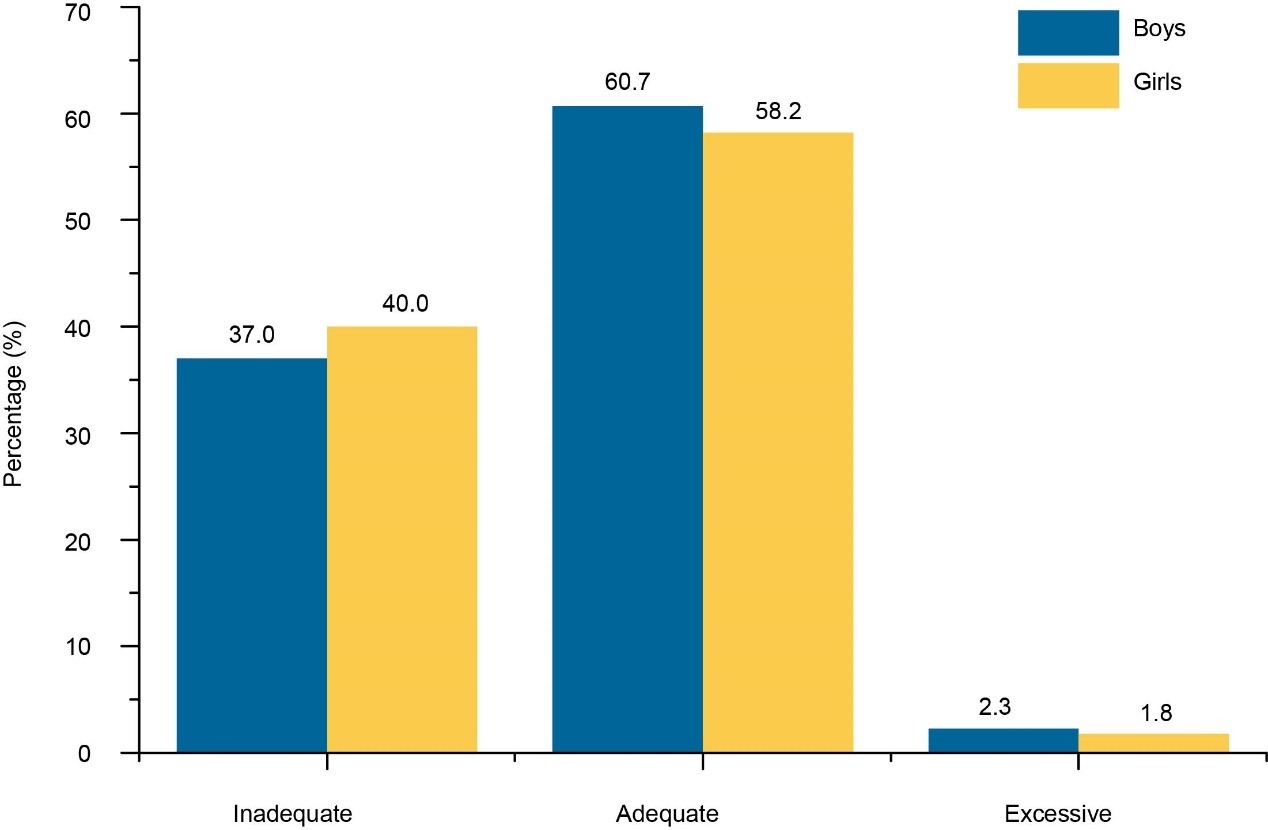


**Supplementary Figure S11.** Sleeping evaluation among students by sex during COVID-19 pandemic.


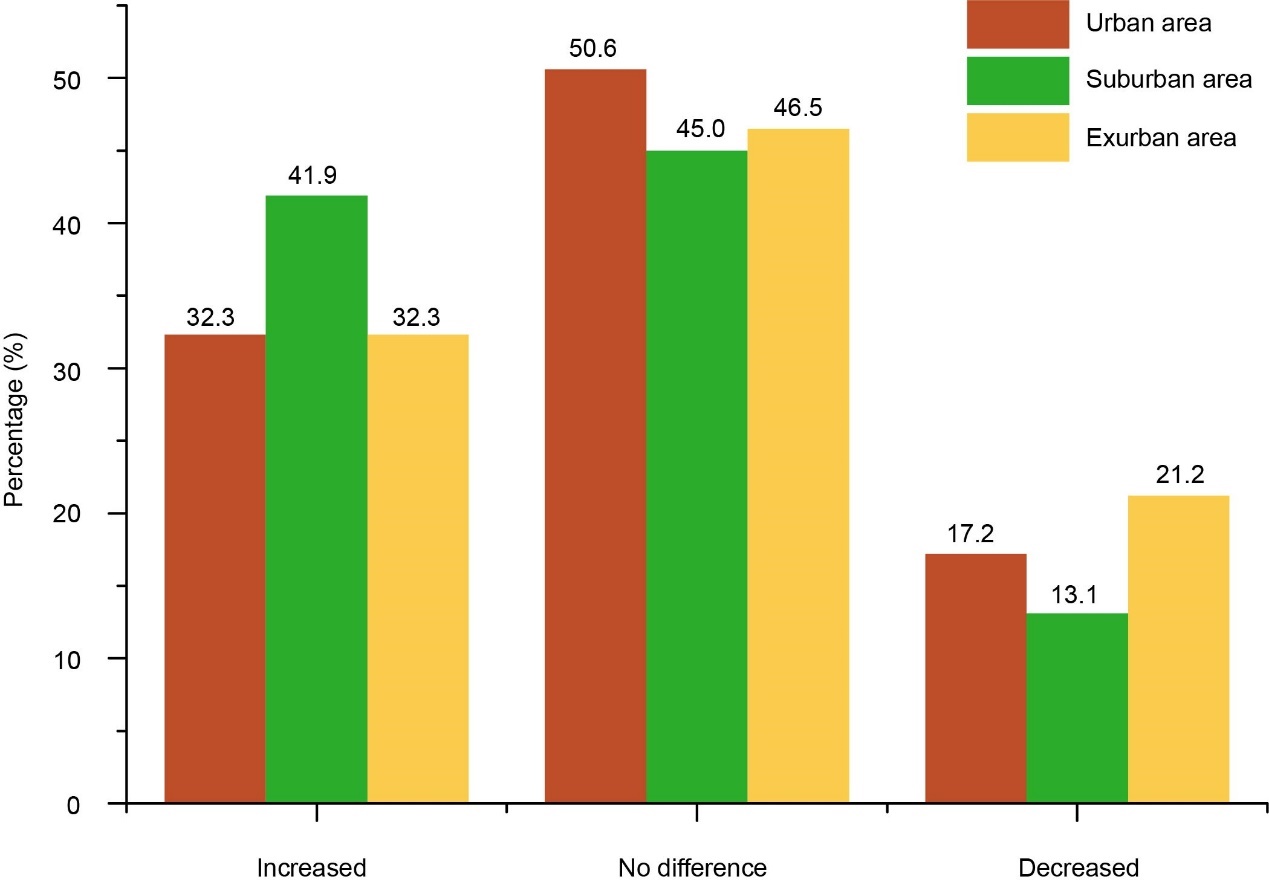


**Supplementary Figure S12.** Changes in sleeping duration among students in various regions during COVID-19 pandemic.


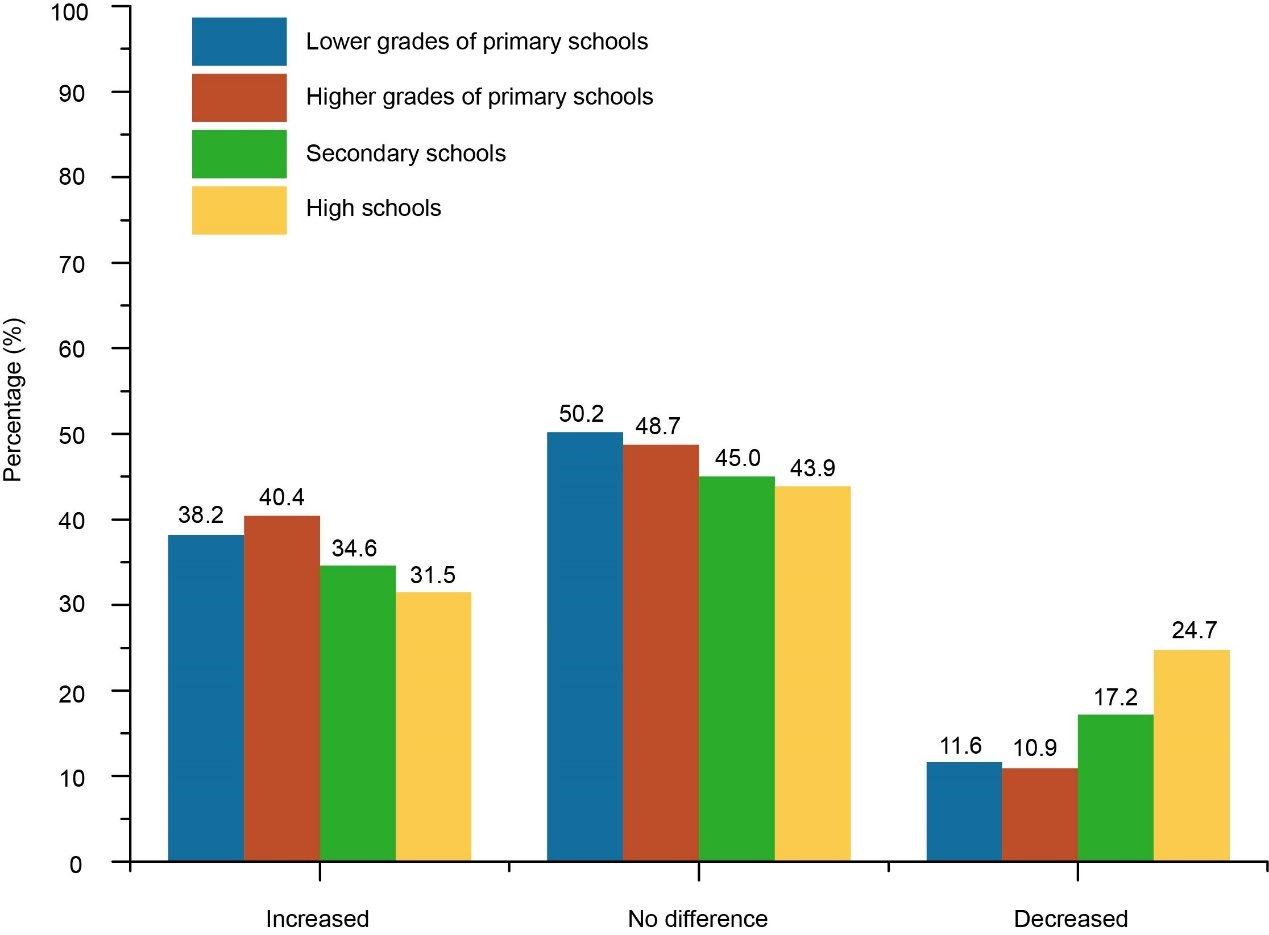


**Supplementary Figure S13.** Changes in sleeping duration among students of different grade categories during COVID-19 pandemic.
